# Gonococcal Vaccines for Controlling *Neisseria gonorrhoeae* in Men Who Have Sex With Men: A Promising Game Changer

**DOI:** 10.1093/infdis/jiab582

**Published:** 2021-12-11

**Authors:** Hannah Christensen, Peter Vickerman

**Affiliations:** Population Health Sciences, University of Bristol, Bristol, United Kingdom

**Keywords:** *Neisseria gonorrhoeae*, vaccine, MSM, model

(See the Major Article by Hui et al, on pages 983–93.)

The disease burden of *Neisseria gonorrhoeae* is high, with the World Health Organization (WHO) estimating there were 82 million new cases globally in 2020 [1]. While gonorrhea is a curable disease, high levels of resistance have developed against a succession of antibiotic treatments with emerging resistance to the last-line option (ceftriaxone or cefixime); dual therapy, principally ceftriaxone plus azithromycin, is currently used in many countries [2]. Although many cases are asymptomatic, complications from infection are serious, especially for women, and can lead to infertility and increased risk of human immunodeficiency virus (HIV) [3]. Taken together, gonorrhea poses a critical threat to public health. The global health sector strategy in 2016 [4] called for a 90% reduction in the incidence of gonorrhea by 2030, but the recent WHO global progress report shows efforts must be accelerated if we hope to meet this goal [1]. Vaccination could offer a game-changing intervention in the battle for control of this sexually transmitted infection (STI). This possibility may now be within reach, given recent evidence that vaccines designed to protect against meningococcal infection, caused by a closely related bacterium *Neisseria meningitidis*, and already in use in some countries, can offer some cross-protection [5, 6]. Specific *N. gonorrhoeae* vaccines are also in the pipeline.

In this issue of *The Journal of Infectious Diseases*, Hui et al [7] present the results from a mathematical modelling study estimating the impact of a vaccination program against *N. gonorrhoeae*, across multiple anatomical sites, in a hypothetical population of 10 000 men who have sex with men (MSM) in Australia. They estimate gonorrhea prevalence could be reduced by 62% within 2 years if a vaccine had 50% efficacy against infection and there is 30% uptake of vaccination in MSM when they present for STI testing. The authors also highlight the importance of boosters for maintaining control, due to the likely relatively short duration of vaccine-induced protection. Hui et al [7] suggest elimination may be possible within 8 years with vaccines of ≥ 50% efficacy and booster doses given on average every 3 years.

Gonorrhea rates in MSM are high, so this group presents an obvious target for vaccination; in European Union/European Economic Area (EEA) countries in 2019, 42% of reported gonorrhea cases were in MSM [8]. The work by Hui et al [7] adds to modelling evidence from England that vaccinating MSM, even with a partially protective vaccine, could have a considerable impact on gonorrhea incidence [9]. Going further, the suggested prospect of elimination of gonorrhea among the MSM community within a decade is enticing. However, the work of Hui et al [7] may be overly optimistic because it is highly dependent on vaccine uptake. The model is set up for a site with very high levels of health contact for MSM globally, assuming 80% of MSM are tested for STIs each year with 30% of them choosing to be vaccinated at each presentation. This level contrasts with Europe where data from 2017 suggests on average only 43% of MSM were tested for STIs in the last year, ranging from 19% in Belgrade to 59% in London [10]. Similarly, rates of HIV testing are also lower in Europe (38%–70% tested in the last year in 2017) [11]. A further consideration is the uniformity in vaccine uptake by MSM. If those who choose not to be vaccinated are more likely to form partnerships with similarly minded individuals, then the reductions in prevalence are less likely to be realized [12].

Currently available control measures for gonorrhea among MSM involve screening and treatment, with provision of these measures varying by setting. In areas where treatment and screening are less well established than in Australia, reducing gonorrhea prevalence may be more challenging than suggested by the model. In addition to the challenge of a likely higher prevalence of gonorrhea in such settings, the lower levels of screening mean lower vaccine uptake in this delivery model, making gonorrhea control through vaccination harder. Increasing prevalence, as observed in many countries worldwide [13], is a further issue. Vaccination against *N. gonorrhoeae* could be the game changer we need in controlling this infection; however, policy makers should consider whether immunization can offer enough to tip the epidemic to enable control or whether more may be needed in terms of current provision of screening and treatment or vaccine delivery to enable this to happen.

The ability of the vaccines to affect transmission of *N. gonorrhoeae* as well as reducing symptoms is an important consideration. A high proportion of *N. gonorrhoeae* infections are asymptomatic, particularly in women. Studies on the vaccines designed to protect against meningococcal B disease have shown they are highly effective against invasive symptomatic disease, but have no real impact on carriage [14, 15]. If a vaccine program were to be introduced in MSM, or other populations, it would be important to monitor asymptomatic infection. If vaccination reduced symptomatic *N. gonorrhoeae* infection leading to fewer MSM seeking treatment, but without decreasing *N. gonorrhoeae* transmission, this property could offset health gains. However, this may not be such an issue if only a minority of MSM attending clinics for STI testing do so because they have symptoms (approximately 14%), as suggested by data from the United States [16]. There may be further consequences of such a vaccine limitation if MSM also have sex with women and pass asymptomatic infections to women. As for all vaccines, if the vaccine is not equally effective against all *N. gonorrhoeae* strains, then vaccination could introduce selective pressure, which could be detrimental and another reason for careful monitoring after vaccine implementation.

While offering vaccines against *N. gonorrhoeae* to MSM attending STI clinics might seem the logical first step to radically reduce cases, this approach excludes many for whom a vaccine could be valuable. An alternative approach may be to vaccinate teenagers, ideally before sexual debut. Due to high rates of meningococcal disease, a vaccine designed to protect against Men B meningococcal disease, 4CMenB, was introduced in South Australia among year 10 students (aged 14–15 years) in 2019, with a time-limited catch-up in those aged 15–21 years. At 2 years postimplementation, they have observed reductions in suspected and confirmed gonorrhea across all settings and a modest vaccine efficacy against disease [6]. It will be interesting to observe the impact of the vaccine on asymptomatic *N. gonorrhoeae* infection as well as symptomatic disease going forward. Indeed, future clinical trials of *N. gonorrhoeae* vaccines need to assess the impact on all *N. gonorrhoeae* infections, and the impact of vaccination by site of infection. Ecological studies of symptomatic cases will be insufficient to appropriately assess vaccine impact. Modelling studies will then be able to appropriately include these factors to estimate long-term impact of the vaccine, and also help inform optimal characteristics for vaccination.

When considering the benefit of vaccination, it is important to consider whether the use of vaccination rather than treatment for gonorrhea control could potentially reduce antimicrobial resistance. This component is complex to appropriately include within a modelling framework but should be a factor in discussions around whether and how to implement programs. Cost-effectiveness calculations may also aid these policy discussions but need to carefully consider the setting and levels of current screening and treatment. The fact that there is a vaccine in use that can reduce gonorrhea could represent a step-change in the way we control gonorrhea; the key now is to understand the generalizability of these model findings to other settings to enable maximum benefit from any immunization program.

## Notes

***Financial support.*** This work was supported by the National Institute for Health Research (NIHR) Health Protection Research Unit in Behavioural Science and Evaluation at the University of Bristol in partnership with Public Health England (support to H. C. and P. V.); and NIHR (grant number CDF-2018-11-ST2-015 Career Development Fellowship to H. C.). P. V. is also supported by the Wellcome Trust (WT 220866/Z/20/Z).

***Potential conflicts of interest.*** H. C. is a Principal Investigator on a project funded by GSK. P. V. is a Principal Investigator on a project funded by Gilead, unrelated to this work. Both authors have submitted the ICMJE Form for Disclosure of Potential Conflicts of Interest. Conflicts that the editors consider relevant to the content of the manuscript have been disclosed.
